# Effects of cryogenic cooling on cutting temperature and surface roughness in turning of AA7075 aluminum alloy

**DOI:** 10.1038/s41598-026-39003-7

**Published:** 2026-02-09

**Authors:** Sadegh Ranjbar, Abolfazl Foorginejad, Sayyed Mohammad Emam, Morvarid Ebadi, Keyvan Shiri

**Affiliations:** 1 Department of Mechanical Engineering, Birjand University of Technology, Birjand, Iran; 2 Department of Mechanical Engineering, Faculty of Engineering, Ardakan University, P.O. Box 184, Ardakan, Iran; 3 Department of Biomedical Engineering, Central Tehran Branch, Islamic Azad University, Tehran, Iran; 4 Department of Mechanical Engineering, University of Tehran, Tehran, Iran

**Keywords:** Dry-Cryogenic machining, Temperature, Surface quality, Engineering, Materials science

## Abstract

The machining process is one of the most widely used methods for converting raw materials into finished products across various industries, owing to its numerous advantages, such as high precision, the capability to produce complex geometries, and superior surface finish. AA7075 aluminum alloy is extensively utilized in the aerospace and automotive sectors due to its exceptional strength-to-weight ratio, moderate corrosion resistance, and fair machinability. During machining operations, the simultaneous imposition of mechanical and thermal loads, coupled with severe plastic deformation, induces changes in the material’s mechanical and metallurgical properties—these alterations must be considered to enhance the component’s performance. Consequently, the surface integrity of the machined part, encompassing metallurgical, mechanical, and topographical aspects, is of paramount importance. Surface roughness, a key indicator of surface texture, directly influences the fatigue life of the component. Employing techniques such as cryogenic cooling with liquid nitrogen can mitigate the temperature in the cutting zone, thereby enhancing surface quality and prolonging component lifespan. In this investigation, the impacts of various dry and cryogenic machining conditions on surface roughness and thermal loads were examined, and the influential parameters were identified.

## Introduction

Nowadays, in the automotive and aerospace industries, the demand for low-density materials that can withstand significant forces has increased. Aluminum alloys are one of the most popular materials for the above conditions due to their high force-to-weight ratio^1^. The unique properties of the alloy, including machinability, heat transfer coefficient, lightness, and wear resistance, AA7075 has become a widely used alloy in the automotive and aerospace industries^2^. It is estimated that approximately 15% of the total value of mechanical components manufactured worldwide is derived from machining operations^3^. In recent years, especially in modern machining industries, the effort to achieve surface quality, dimensional accuracy, high production rates, and cost savings has become very important. In the turning process, surface quality is measured by the surface roughness deviation, which is an important parameter that the customer will consider for the turned parts^4^. The three machining parameters—cutting speed, feed rate, and depth of cut—were selected because they are widely recognized as the most influential input variables governing thermal loads, chip formation mechanisms, and the resulting surface integrity in turning processes. Numerous studies have demonstrated that changes in these parameters directly affect heat generation at the tool–workpiece interface, the stability of chip removal, and the final surface texture of machined components^5,6^. Today, manufacturing and production processes, including machining, are widely used in manufacturing industries to convert raw materials into finished products due to their many advantages. During these processes, severe mechanical-thermal loads are generated in the material and can cause microstructural changes, the formation of microcracks and residual stresses in the final product^7^, and [for this reason, the surface health after machining in hard-to-cut materials is an important issue and has surface texture (roughness and surface quality), metallurgical (microstructural changes, formation of white and black layers, phase change) and mechanical (residual stress and plastic deformation) dimensions^8^. Recent studies have shown that machining and cutting processes can exhibit non-linear or counterintuitive trends in surface quality due to complex interactions between thermal loads, material softening, and process dynamics^9,10^. Thermal loads generated during the process are one of the important factors in examining the surface health after machining and mainly cause the formation of tensile residual stresses in the material and microstructural changes such as the formation of white and black layers in steel alloys^11^. The roughness of the machined surface is one of the components of the surface texture and has a significant impact on the life and quality of the part. In addition, thermal loads generated during the machining process can increase the amount of surface roughness. Therefore, a high level of surface roughness reduces fatigue life and increases the likelihood of microcracks forming and spreading on the machined surface^12^. On the other hand, changes caused by thermal loads can reduce the life of the tool and the part and also increase production costs. Therefore, using methods to reduce thermal loads and microstructural changes can have a significant impact on increasing the performance and quality of parts^13^. Some studies have shown that machining-induced surface integrity can exhibit complex and sometimes counterintuitive behavior due to the interaction between thermal loads, chip formation mechanisms, and material responses. For example, investigations on Ti6Al4V components produced by additive manufacturing have demonstrated that machining parameters can significantly affect surface morphology, burr formation, and microstructural stability, highlighting the non-linear nature of machining responses^14^. Similarly, research on the effect of machining processes on the physical and surface characteristics of powder-bed-fusion Ti6Al4V parts has shown that cutting conditions may lead to unexpected variations in roughness and material behavior due to localized thermal gradients, chip adhesion, or temperature-dependent brittleness^15^. These findings indicate that machining outcomes—particularly surface roughness—may not always follow linear or intuitive trends, and can vary depending on dynamic thermal–mechanical interactions. In recent years, various strategies, including the use of coolant, high coolant pressure, and nitrogen gas, have been implemented to improve the machining process. In the coolant method, liquid nitrogen is sprayed onto the contact area between the tool and the workpiece during the chip removal process, causing a significant reduction in temperature in the cutting zone. As a result, microstructural changes, residual tensile stresses, and the heat-affected zone are also reduced, and the performance of the final product is improved^16–18^. Dananchezian and colleagues^19^ found in their research that the use of coolant can reduce the temperature of the cutting zone by up to 50%. In addition to the above, the use of coolant has a significant effect on increasing the life of the tool. Due to these advantages, researchers widely use various cooling methods to increase the quality and efficiency of products^20^. A number of studies conducted by other researchers are described below.

Mithuramanan et al.^5^ investigated the performance of cryogenics as a cutting fluid in 7075 aluminum alloy and compared the results with traditional (wet) machining in terms of machining temperature, force reduction and surface roughness reduction. Amber low^21^ investigated the effect of machining parameters on the surface health of steel alloy. AISI 52,100 was investigated. His results show that with increasing cutting speed, the number of thermal loads during the operation increases. In another study, during the experiments of Sivaiah et al.^22^ on the temperature and roughness of the machined surface of steel alloys, it was reported that without the use of cooling, the temperature of the machining area and the surface roughness increase significantly, but when cooling is used, the temperature and roughness values ​​decrease. Kumar et al.^23^ studied the effect of the tool tip and the hardness of the workpiece on the number of thermal loads generated during the machining process of AISI 4340 Steel. In another study, Abdul Karim et al.^24^ studied the effect of machining parameters on the thermal loads generated during the machining process of AISI 1045 steel and found that the temperature of the machining area increases mainly with increasing depth of cut and speed. In the studies of Tang et al.^25,26^ on the effect of the initial hardness of the workpiece on the quality of the machined surface, it was found that as the initial hardness of the workpiece increases, the roughness increases. They also found that increasing the hardness mainly reduces thermal loads.

Given the increasing demand for lightweight and high-performance components, aluminum alloys have become essential materials across automotive, aerospace, and other advanced manufacturing industries. Among them, AA7075 is widely utilized due to its high strength-to-weight ratio and favorable mechanical properties. Despite its extensive industrial application, limited studies have comprehensively examined the surface quality and thermal loads generated during machining of AA7075, particularly under dry and cryogenic conditions. Understanding these effects is crucial for improving product reliability, extending component lifespan, and optimizing machining performance. Given the frequency of use of this material, the surface quality and microstructural changes caused by the thermal loads produced during the machining process have a significant impact on the performance of the manufactured part. This indicates the importance of studying this issue. Given the advantages of using liquid nitrogen as a coolant, the effects of using this method on the health of the final product require further investigation. In the present study, the effect of changing machining parameters when using and not using a coolant was studied simultaneously. Finally, the effect of changing each of the parameters on the thermal loads and surface roughness produced during the process was investigated and its results were extracted. In this regard, first 7 tests were conducted under dry conditions and 7 tests under cool conditions of the work. A thermal camera was used to investigate the thermal loads created during the process. Then, using a roughness meter, the average roughness of the machined surface was measured. Finally, the effect of each parameter on surface quality and thermal loads was extracted and reported.

## Materials and methods

In order to accurately investigate the effect of machining conditions on the thermal behavior and surface quality of AA7075 aluminum alloy, a series of controlled experiments were conducted in both dry and cryogenic conditions. The aim of this section is to provide a precise basis for analyzing the effect of machining parameters on surface roughness and cutting zone temperature; factors that directly affect surface integrity, fatigue life, and final performance of the part. The experiments were conducted on cylindrical parts with a diameter of 23 mm and a length of 60 mm, which were prepared from AA7075 aluminum alloy with a standard chemical composition as shown in Table 1.

**Table 1:** Chemical composition of aluminum alloy AA7075.

Elements7075	Aluminum	Silicon	Copper	Manganese	Magnesium	Chromium	Zinc
Composition %	Bal.	0.4	0.098	1.41	0.055	2.33	5.95

Machining was performed using a TN50D lathe, in the spindle speed range up to 1000 rpm. The cutting tool used was a tungsten carbide insert type TNMG11510 with a tip radius of 0.4 mm, which was considered a suitable choice for this process due to its high hardness and wear resistance. To evaluate the machining conditions, seven different combinations of key parameters including cutting speed (Vc), feed rate (f) and depth of cut (ap) were considered in both dry and cryogenic cooling modes. In the cooling mode, liquid nitrogen was sprayed directly into the tool-part contact area to objectively observe its effect on reducing local temperature and surface changes. The operating parameters of each test are presented in Table 2. To evaluate the thermal behavior and surface quality, each machining test was performed once for every parameter combination; however, repeated measurements were carried out to ensure reliability. Surface roughness was measured using a PCE RT 2200 roughness tester at three different positions on each machined sample, and the mean value was reported as the Ra parameter. For temperature assessment, a thermal camera with calibrated emissivity and controlled relative humidity was positioned at a fixed distance from the cutting zone. The camera continuously monitored the cutting area during each machining pass, and the maximum stabilized temperature was extracted for analysis.

**Table 2:** Applied machining parameters during the experimental process.

Test number	Vc (rpm)	ap (mm)	af (mm/rev)
1	710	0.4	0.16
2	500	0.4	0.16
3	1000	0.4	0.16
4	710	0.4	0.08
5	710	0.4	0.22
6	710	0.2	0.16
7	710	0.6	0.16

These experiments have been designed with high precision and purpose to not only determine the effect of each parameter individually, but also to examine the interaction between them under different operating conditions (dry and cold). This scientific and systematic methodology provides the necessary basis for in-depth analysis in the results section and increases the validity of the research findings.

**Fig. 1:**
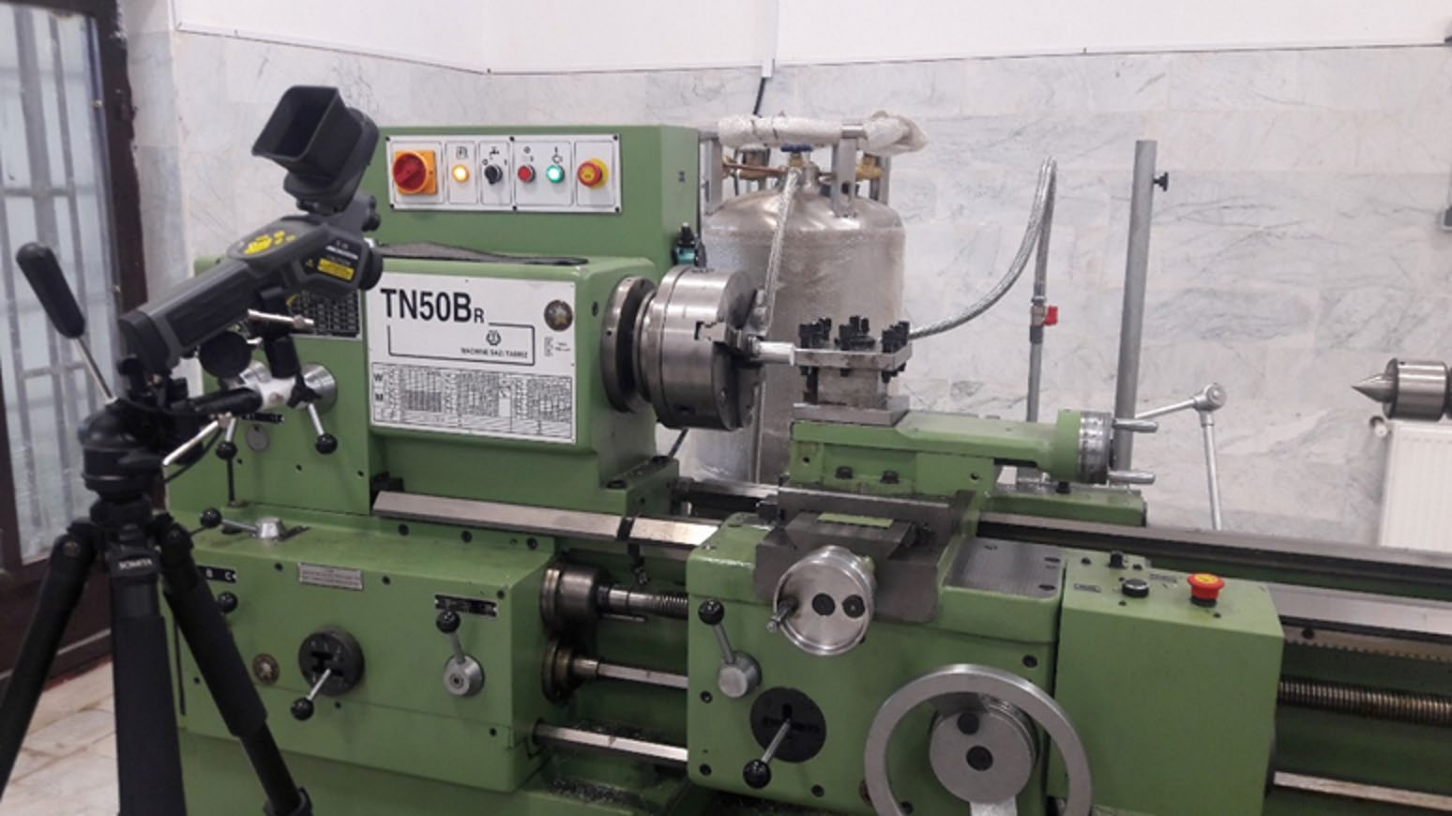
Machine tool equipped with a liquid nitrogen cooling tank.

A detailed evaluation of temperature distribution, chip formation behavior, and surface quality in the machining of AA7075 aluminum alloy was carried out through Test No. 7 under both dry and cryogenic conditions. The experiments were performed on cylindrical specimens with a diameter of 23 mm and a length of 60 mm using a TN50D lathe with a maximum spindle speed of 1000 rpm, as shown in Fig. 1. Chip flow characteristics were examined using tungsten carbide inserts (TNMG11510) with a tool-tip radius of 0.4 mm. To analyze the influence of machining variables on thermal loads and surface finish, the cutting parameters—including feed rate, depth of cut, and cutting speed—were systematically varied under both dry and cryogenic cooling environments. Figure 1 also illustrates the cryogenic setup, where liquid nitrogen is supplied through a dedicated reservoir and directed toward the tool–workpiece interface. The direct injection of liquid nitrogen into the cutting zone significantly reduces localized temperatures and contributes to improving the resulting surface quality.

## Discussion and results

In this section, the effect of various machining parameters such as cutting speed and depth of cut on the cutting zone temperature and surface roughness in dry and cold conditions has been investigated. For a more detailed analysis, comparative graphs have been prepared and quantitative analyses of the test results have been presented.

### Comparison of shear temperature and surface roughness in dry and cryogenic conditions

According to the results, cryogenic cooling produced a substantial reduction in the cutting-zone temperature compared to dry machining. In several test conditions, the temperature difference between dry and cryogenic machining exceeded 50 °C, demonstrating the strong thermal-suppression capability of liquid nitrogen. This considerable temperature drop significantly limits heat accumulation in the cutting area and contributes to improvements in surface quality and metallurgical stability of the machined surface.

### Effect of shear rate on temperature and surface roughness (in dry conditions)

With increasing cutting speed from 500 to 1000 rpm, the cutting-zone temperature rose from approximately 94 to 108 °C, accompanied by an increase in surface roughness from about 2.0 to 2.5 μm. This behavior can be attributed to the higher frictional energy and heat generation at the tool–workpiece interface at elevated speeds. As a result, operating at high cutting speeds without effective thermal control may degrade surface quality and reduce the service life of the machined component.

### Effect of cutting depth on cutting zone temperature and surface roughness

To analyze the effect of cutting depth on thermal variables and surface quality, separate graphs were drawn for cutting zone temperature and surface roughness. This separation was done to more accurately examine the behavior of each parameter against changes in cutting depth. The results show that, with increasing cutting depth from 0.2 to 0.6 mm, the temperature of the cutting zone has increased significantly; so that the temperature has reached from about 102 ° C to more than 139 ° C. This increasing trend can be attributed to the increase in the volume of chip removal, a larger contact area between the tool and the workpiece, and consequently increased friction and heat generation. The increase in temperature in this range indicates the high sensitivity of the process to the depth of cut parameter in dry conditions.

The results show that with increasing cutting depth, the surface roughness also increases and ranges from 1.9 to 2.4 μm. This increase in roughness may be due to two factors: first, higher mechanical load on the tool, which causes changes in chip formation, and second, increased local temperature, which can cause local softening of the material and reduce the controllability of the chip removal process.

Overall, the results show a direct relationship between depth of cut and both output parameters (temperature and surface roughness). Therefore, in precision machining processes, controlling and optimizing depth of cut is of particular importance as one of the key factors in ensuring surface quality and reducing thermal stresses.

Increasing the cutting depth leads to an increase in mechanical and thermal load in the cutting zone, which causes an increase in temperature and surface roughness. The results have shown that by increasing the cutting depth from 0.2 to 0.6 mm, the cutting zone temperature increased by about 80 degrees and the surface roughness increased by about 0.5 μm.

In the following, an experimental study of thermal loads and surface quality after machining under different cutting conditions is carried out. When machining the samples under dry and cold conditions with different cutting speeds, cutting depths and feed rates, the thermal loads generated at the machining site were measured by a thermal camera. After machining the samples by a roughness meter roughness Tester PCE RT 2200 Roughness was measured at three points and the average was reported. Then, the effect of each machining condition on the number of thermal loads generated in the shear zone and the surface roughness after machining were investigated and compared with each other.

### Thermal load check

**Fig. 2:**
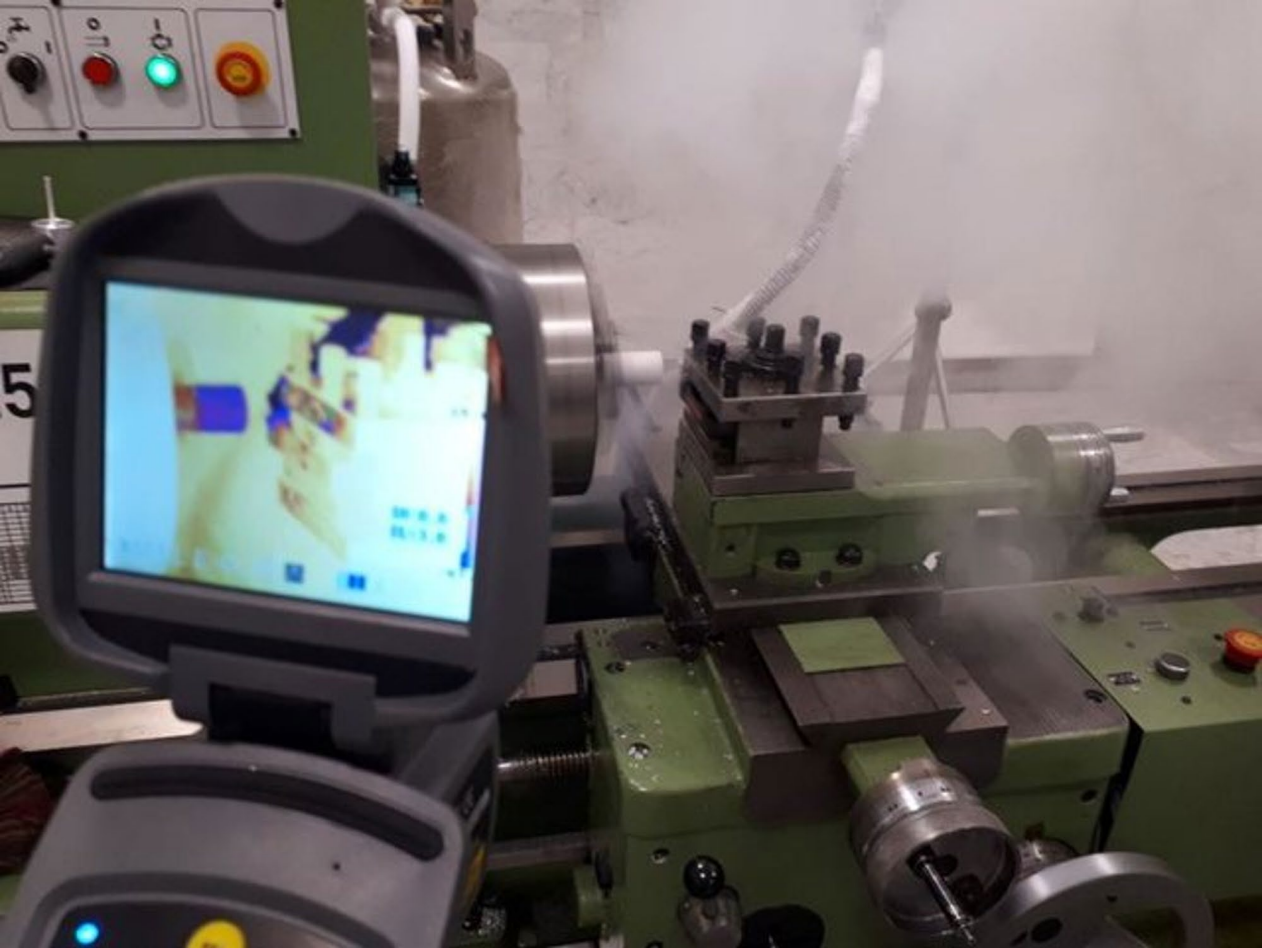
Thermal camera setup for measuring thermal loads generated in the machining zone.

The contact between the tool and the workpiece during the machining operation mainly causes plastic deformation and thermal loads in the cutting zone. The thermal loads generated during the machining process cause microstructural changes, tensile residual stresses and premature fatigue failure in the material^26^. Due to the importance of these changes to the final product, using methods that reduce the temperature in the cutting zone can improve the reliability, quality, and performance of the part^16^. Liquid nitrogen spraying in the cutting zone is an effective strategy to reduce the thermal loads generated during the process. In this study, a number of experiments were conducted to compare the thermal loads generated in the machining area under dry and cold conditions. A thermal camera was used to measure the heat generated in the cutting zone during the operation, and the following parameters were recorded: Emissivity = 0.25 and Humidity = 40%was considered. For this purpose, a thermal camera was placed at a distance of 1 m from the machine tool and the temperature distribution in the machining area was measured during the process. Figure 2 shows the spraying of liquid nitrogen into the cutting area, the thermal camera, and its placement.

**Fig. 3:**
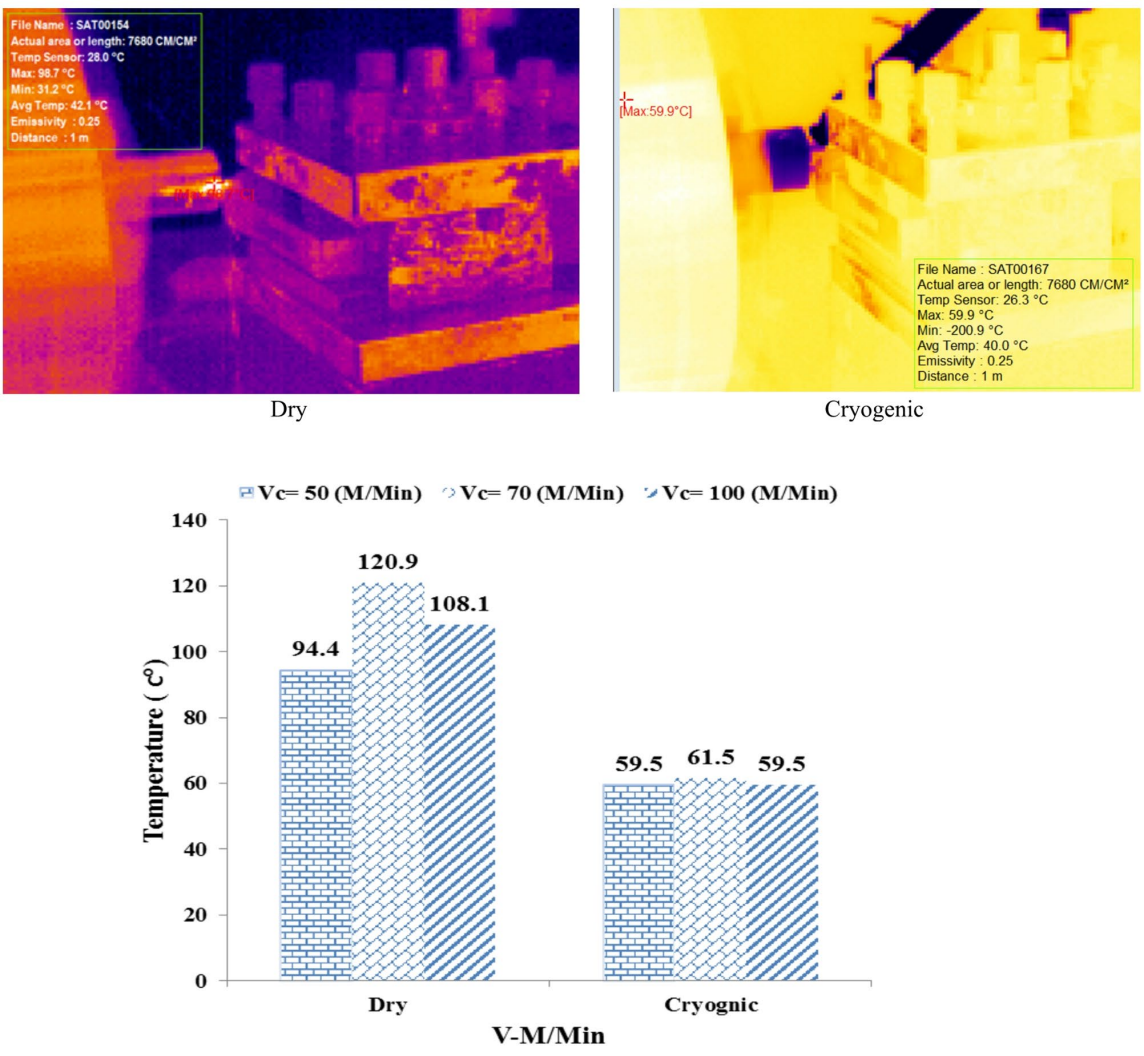
Thermal camera images of the cutting zone during machining under cryogenic and dry conditions.

In Fig. 3 Example of images captured by a thermal camera of the cutting zone during the machining process under different conditions. The images on the left are from cryogenic machining using liquid nitrogen and show a significant decrease in the temperature of the cutting zone (the red and yellow area is more limited), while the images on the right are from dry machining, where the expansion of the high-temperature zones (red and orange) is clearly visible. This thermal difference indicates the effective role of cryogenic cooling in suppressing heat generation and preventing the occurrence of thermal stresses and unwanted microstructural changes in the workpiece.

Figure 3 provides a detailed comparison between the cutting zone temperature in both dry and cryogenic machining conditions at three different cutting speed values. (Vc) means 50, 70 and 100 m per minute.

Dry conditions (Dry): In the dry state, with increasing shear rate, the temperature of the shear zone increased continuously:

Vc = 50 Temperature = 94.4 °C.

Vc = 70 Temperature = 120.9 °C.

Vc = 100 Temperature = 108.1 °C.

The temperature increase in this case is due to the intense friction between the tool and the workpiece and the lack of an effective heat dissipation system. These high temperatures not only negatively affect the mechanical and metallurgical properties of the workpiece, but also accelerate tool wear.

Cryogenic conditions (Cryogenic): In cryogenic conditions, despite the change in shear rate, the temperature remained almost constant and controlled:

Vc = 50 Temperature = 59.5 °C.

Vc = 70 Temperature = 61.5 °C.

Vc = 100 Temperature = 59.5 °C.

**Fig. 4:**
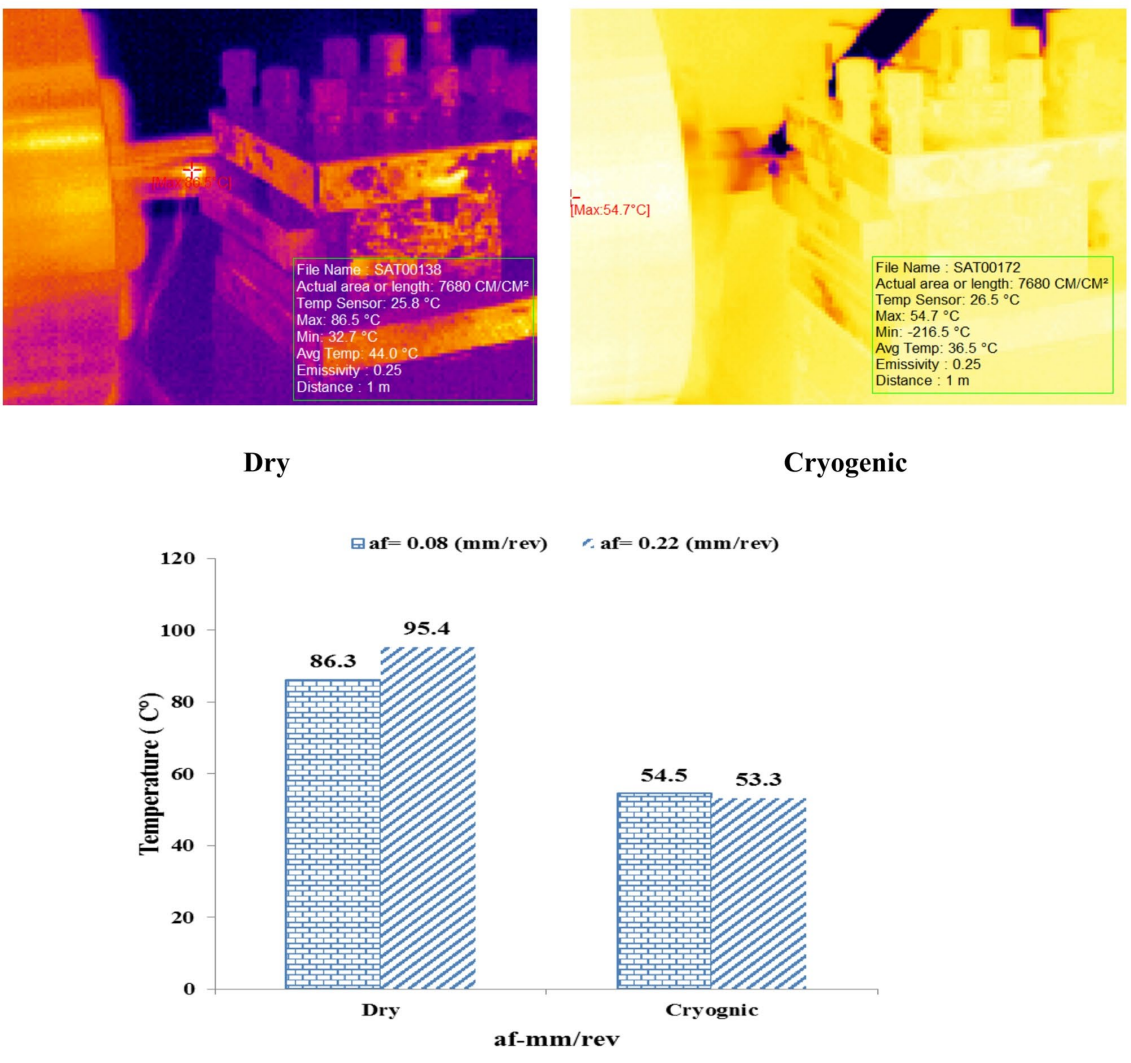
Comparison of cutting-zone temperature under dry and cryogenic machining at two feed rates (af = 0.08 and 0.22 mm/rev).

This temperature stability demonstrates the highly effective performance of liquid nitrogen in removing heat generated in the cutting zone. In particular, the lack of temperature increase at high speeds (unlike dry conditions) makes this method even more important in machining sensitive or heat-retentive parts. Figure 4 shows the effect of two different values ​​of the forward rate. (af = 0.08 and 0.22 mm/rev) Displays the temperature of the cutting zone in two modes: dry machining and cryogenic machining.

#### In dry conditions (Dry)

By increasing the progress rate from 0.08 to 0.22 mm/rev, cutting zone temperature from 86.3 °C To 95.4 °C has increased. This temperature increase is justified because a higher feed rate means a larger volume of material is introduced into the cutting zone per tool revolution, resulting in more thermal energy being generated in the process.

#### In cryogenic conditions (Cryogenic)

**Fig. 5:**
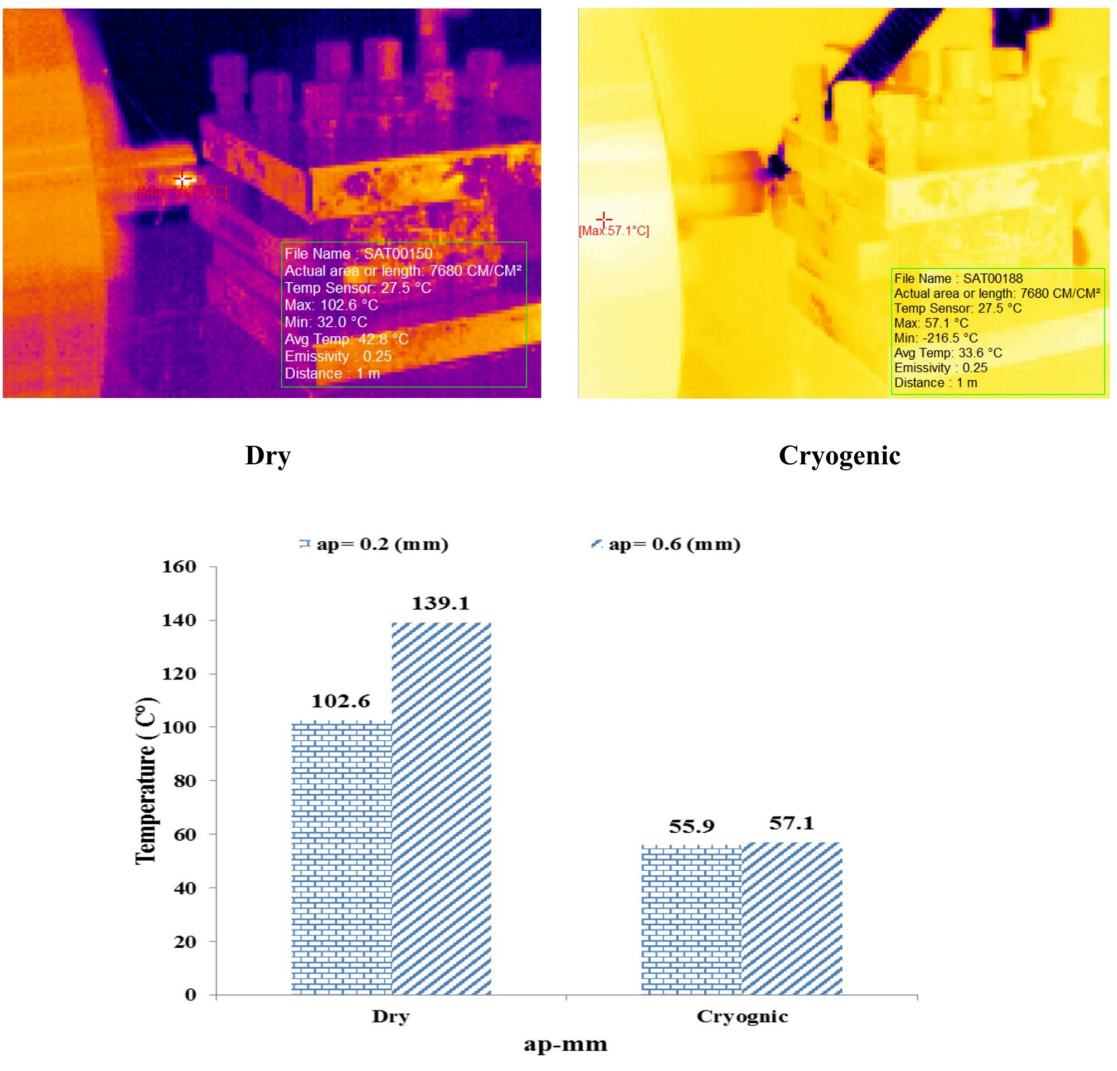
Temperature variations in the cutting zone at two cutting-depth (ap = 0.2 and 0.6 mm).

Temperature at advance rates of 0.08 and 0.22 mm/rev respectively 54.5 °C and 53.3 °C It has been. The very small difference between these two values ​​indicates that the use of liquid nitrogen has thermally stabilized the process and largely neutralized the effect of changing the feed rate. Figure 5 shows the temperature changes in the cutting zone for two different values ​​of cutting depth (ap = 0.2 and 0.6 mm) It is in dry machining and cryogenic machining conditions.

#### In dry condition (Dry)

As the cutting depth increases from 0.2 to 0.6 mm, the temperature102.6 °C to 139.1 °C has increased. This significant increase is due to the greater volume of material involved in the removal process and the increased contact area of ​​the tool with the workpiece, which leads to greater friction and higher heat generation in the cutting area.

#### In cryogenic mode (Cryogenic)

**Fig. 6:**
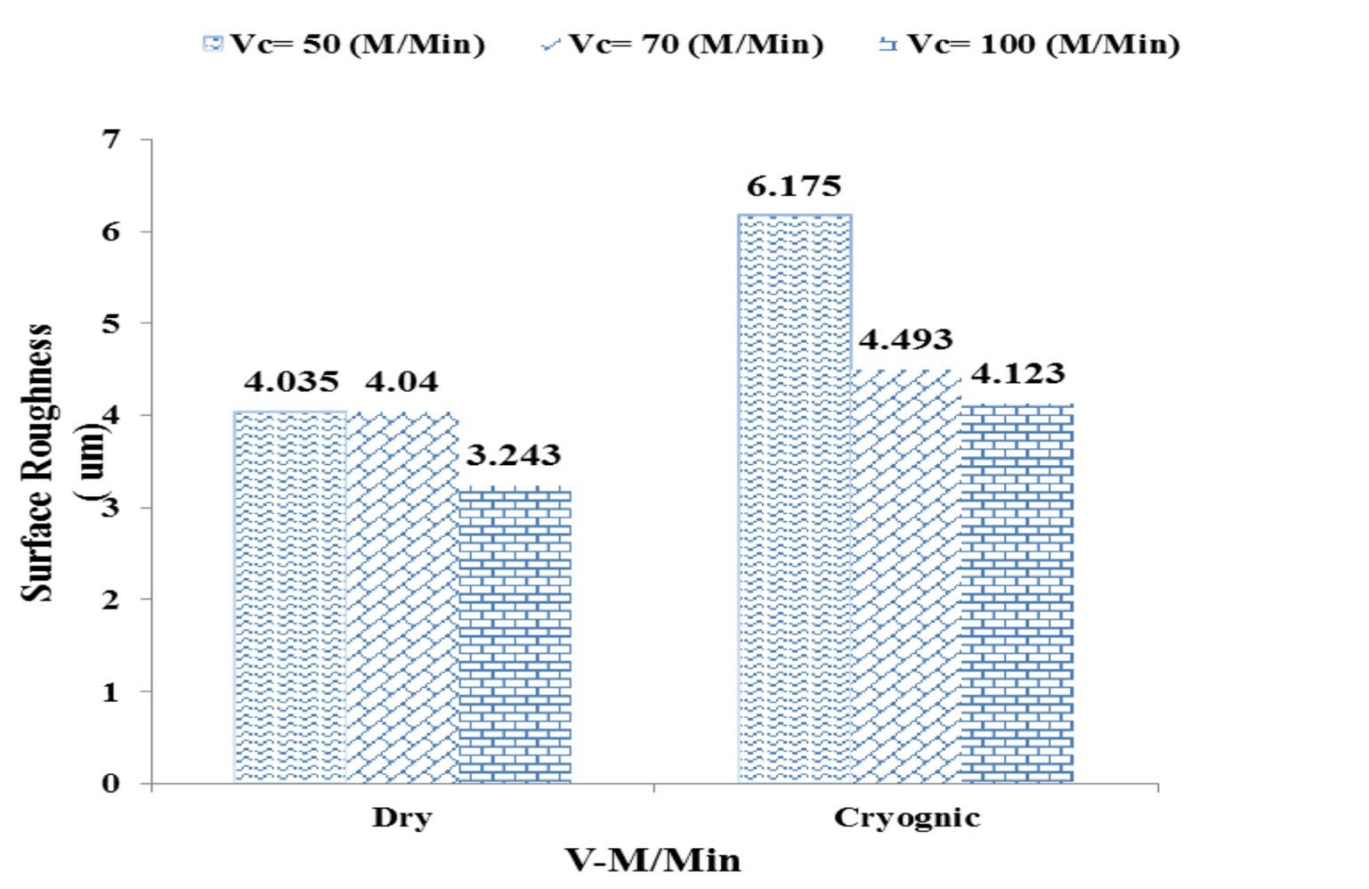
Comparison of surface roughness at three cutting speeds (Vc = 50, 70, and 100 m/min).

In this case, increasing the depth of cut only causes a slight change in temperature from55.9 °C To 57.1 °C It has been. This thermal stability demonstrates the effective performance of the cryogenic cooling system in rapidly dissipating heat and limiting the thermal impact of the process parameters. Figure 6 examines and compares surface roughness. (Ra) for three different values ​​of shear rate (Vc = 50, 70 and 100 m/min Collar) in two dry and cryogenic machining conditions. The results obtained show a significant and contradictory behavior between the two conditions.

In dry conditions (Dry):

By increasing the shear rate from 50 to 100 m/min, surface roughness decreased:

Vc = 50 → Ra = 4.035 μm.

Vc = 70 → Ra = 4.040 μm.

Vc = 100 → Ra = 3.243 μm.

This reduction in roughness can be attributed to smoother stock removal and reduced friction at high speeds, which has improved surface quality in the dry state.

In cryogenic conditions (Cryogenic):

Unlike the dry state, in cryogenic conditions the surface roughness was higher at lower speeds and decreased with increasing shear rate.:

Vc = 50 → Ra = 6.175 μm.

Vc = 70 → Ra = 4.493 μm.

Vc = 100 → Ra = 4.123 μm.

**Fig. 7:**
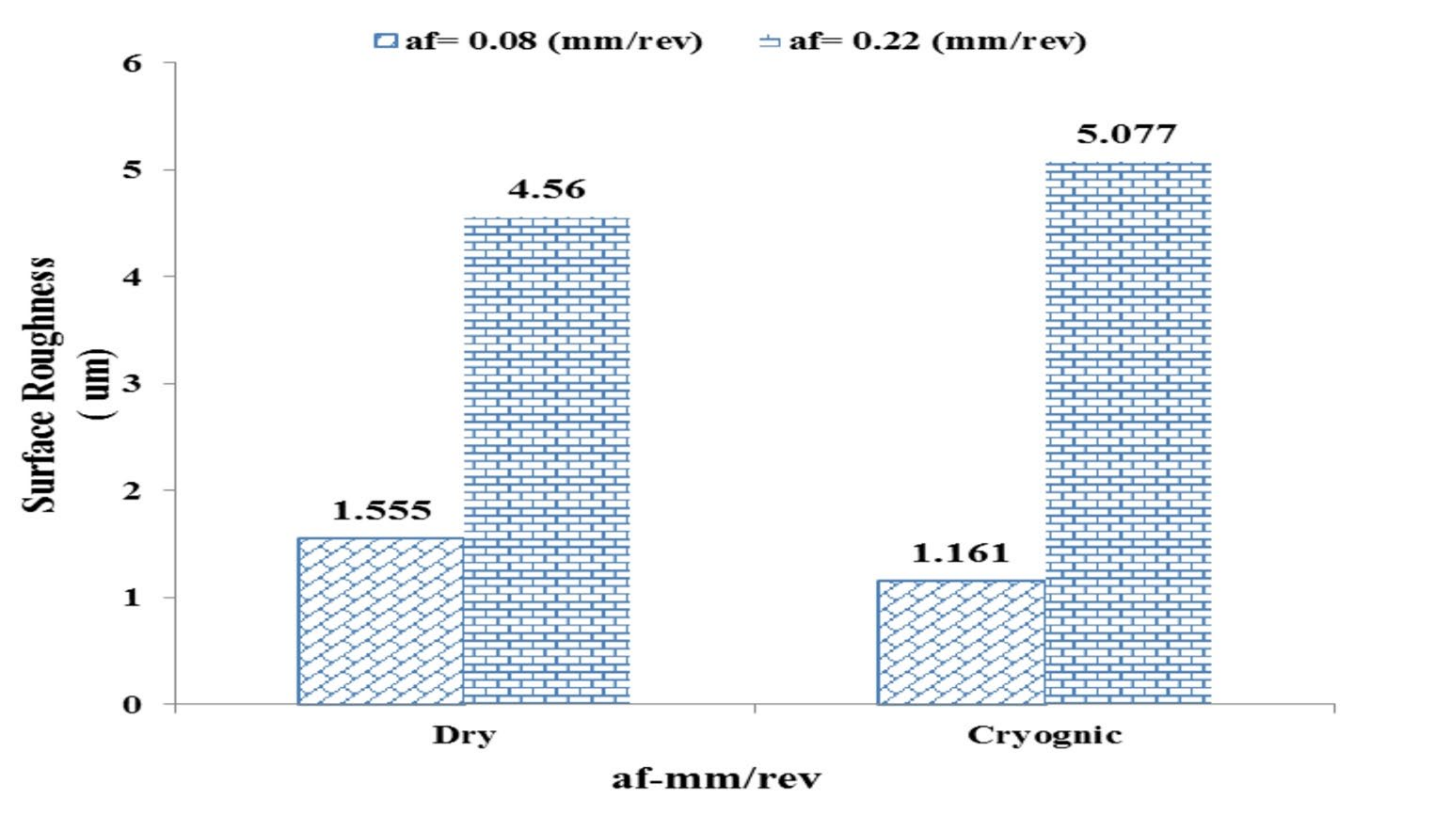
Effect of feed rate (af) on surface roughness under dry and cryogenic machining.

The increase in roughness at low speed (Vc = 50) can be attributed to the disruption of stable chip removal conditions due to more severe local freezing and possible chip adhesion to the tool. At higher speeds, the surface roughness decreases with an increase in the relative contact temperature and smoother chip removal. Investigation of the surface roughness of machined parts under dry and cryogenic conditions showed that the cutting speed (Vc) plays a key role in determining the final surface quality. Under dry conditions, increasing the cutting speed systematically led to a decrease in surface roughness, indicating more stable chip removal, reduced friction, and better process control at higher speeds. In contrast, under cryogenic conditions, the surface roughness at low speed (Vc = 50 m/min) was significantly higher, which could be due to excessive cooling and rapid surface freezing, leading to the formation of inhomogeneous and rough chips. As the speed increased, the surface roughness decreased and reached values ​​close to the dry state, but there were still fluctuations, which emphasizes the need for careful selection of parameters in cryogenic machining. Figure 7 shows that the rate of progress(af) has a direct and strong effect on surface roughness, but the intensity and pattern of this effect varies depending on the cooling conditions.

#### In dry conditions (Dry)

At a low feed rate (af = 0.08 mm/rev), the measured surface roughness was 1.555 μm, which indicates an acceptable and relatively smooth finish. However, increasing the feed rate to 0.22 mm/rev resulted in a substantial rise in surface roughness to 4.56 μm. This deterioration in surface quality can be attributed to the formation of thicker chips, increased cutting vibrations, and higher friction at the tool–workpiece interface, all of which contribute to intensified surface irregularities.

#### In cryogenic conditions (Cryogenic)

**Fig. 8:**
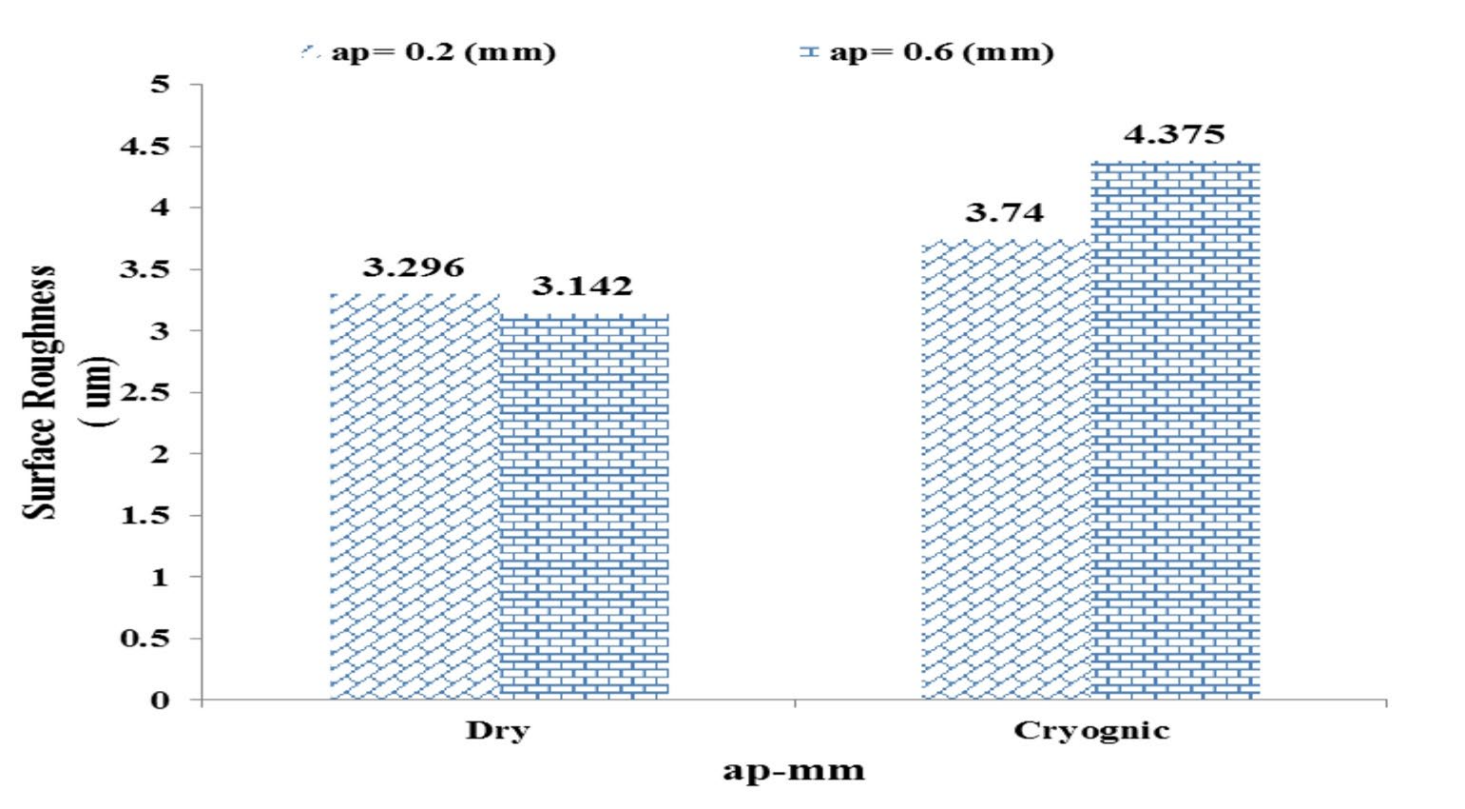
Effect of cutting depth (ap) on surface roughness under dry and cryogenic machining.

At low feed rates, the measured surface roughness under cryogenic cooling was 1.161 μm, which is even lower than the value obtained in dry machining. This indicates the effective performance of liquid nitrogen in stabilizing the cutting process and enhancing surface quality at lower material removal rates. However, when the feed rate increased to af = 0.22 mm/rev, the surface roughness rose markedly to 5.077 μm—exceeding the corresponding value recorded under dry conditions. This behavior suggests that at higher feed rates, the intense cooling effect may induce local embrittlement and promote unstable or irregular chip formation, ultimately leading to increased roughness. Figure 8 illustrates the influence of increasing the cutting depth from 0.2 to 0.6 mm on surface roughness under both dry and cryogenic conditions.

#### In dry conditions (Dry)

By increasing the cutting depth from 0.2 to 0.6 mm, the surface roughness has decreased from 3.296 to 3.142 μm. This relatively small and unusual decrease could be due to the greater stability of the process at greater depths and the reduction of surface vibrations. However, the difference between the two values ​​is small and may be within the measurement error.

#### In cryogenic conditions (Cryogenic)

Surface roughness with increasing cutting depth from 0.2 to 0.6 mm increased significantly from 3.74 To 4.375 The micrometer has arrived. This increasing trend can be attributed to the increase in chip removal volume along with intense cooling, which increases the likelihood of unstable chips, local adhesion, and greater impact on the part surface.

## Conclusion

In terms of industrial applicability, liquid nitrogen cooling offers several potential advantages compared to conventional coolant systems. From a sustainability perspective, LN₂ is an environmentally benign medium that evaporates into nitrogen gas, leaving no chemical residue, eliminating disposal requirements, and avoiding contamination of machined parts or the workplace. Safety can be effectively managed through proper insulation, controlled delivery systems, and operator training, which are standard practices in industries that already use cryogenic substances.

In this study, the effect of using cryogenic cooling with liquid nitrogen on the turning process of 7075 aluminum alloy was investigated. Experimental results showed that cryogenic cooling leads to a significant reduction in the cutting zone temperature, improved surface roughness, and increased final part quality. Recent studies have also confirmed that cryogenic cooling can reduce surface roughness by up to 23%. Reduce and improve tool performance. In addition, the use of cryogenic cooling has a positive effect on reducing residual stresses in machined parts. Research has shown that this method can significantly reduce residual stresses, which leads to improved mechanical properties and increased fatigue life of party. Also, cryogenic cooling with liquid nitrogen is recommended as a sustainable and effective method in machining processes of light alloys such as 7075 due to its favorable environmental characteristics and lack of pollution. Recent studies have also emphasized the environmental and economic benefits of this method.

Overall, the results of this research and related studies show that the use of cryogenic cooling with liquid nitrogen can effectively improve the surface quality, mechanical properties, and stability of the machining process of 7075 aluminum alloy. Surface roughness is one of the dimensions of surface texture and is effective on the fatigue life of the material. Considering the above, using methods that reduce the temperature in the cutting zone can increase the reliability, quality and life of the final product. One of these strategies is the use of coolant, in which the temperature is significantly reduced by spraying liquid nitrogen in the cutting zone. In the present study, the changes in thermal loads and surface roughness for different cutting parameters under dry and cryogenic conditions were measured and compared with each other. Finally, according to the results obtained, the most effective machining parameters on thermal loads and surface quality were determined.

The present study has certain limitations that should be considered when interpreting the results. The experiments were conducted at a laboratory scale with a limited number of parameter combinations, and the focus was restricted to temperature variation and surface roughness. Tool wear progression, chip morphology evolution, and the long-term stability of the cutting tool under cryogenic cooling were not monitored, which may influence both surface quality and thermal behavior. In addition, fatigue performance and mechanical durability of the machined samples were not evaluated, even though these factors are directly affected by surface integrity. Future studies should therefore extend the current work by incorporating systematic tool wear analysis, detailed metallurgical characterization, and mechanical testing such as hardness, tensile strength, and fatigue life evaluation. Expanding the experimental matrix and validating the results under industrial machining conditions will further enhance the applicability and robustness of the findings.

## Data Availability

All data generated or analyzed during this study are included in this published article.
